# In Vitro Degradation of Borosilicate Bioactive Glass and Poly(l-lactide-co-ε-caprolactone) Composite Scaffolds

**DOI:** 10.3390/ma10111274

**Published:** 2017-11-06

**Authors:** Jenna Tainio, Kaarlo Paakinaho, Niina Ahola, Markus Hannula, Jari Hyttinen, Minna Kellomäki, Jonathan Massera

**Affiliations:** BioMediTech Institute and Faculty of Biomedical Sciences and Engineering, Korkeakoulunkatu 3, 33720 Tampere, Finland; jenna.tainio@tut.fi (J.T.); kaarlo.paakinaho@tut.fi (K.P.); niina.ahola@iki.fi (N.A.); markus.hannula@tut.fi (M.H.); jari.hyttinen@tut.fi (J.H.); minna.kellomaki@tut.fi (M.K.)

**Keywords:** bioactive glass, PLCL copolymer, composite, scaffolds, hydroxyapatite

## Abstract

Composite scaffolds were obtained by mixing various amounts (10, 30 and 50 weight % [wt %]) of borosilicate bioactive glass and poly(l-lactide-co-ε-caprolactone) (PLCL) copolymer. The composites were foamed using supercritical CO_2_. An increase in the glass content led to a decrease in the pore size and density. In vitro dissolution/reaction test was performed in simulated body fluid. As a function of immersion time, the solution pH increased due to the glass dissolution. This was further supported by the increasing amount of Ca in the immersing solution with increasing immersion time and glass content. Furthermore, the change in scaffold mass was significantly greater with increasing the glass content in the scaffold. However, only the scaffolds containing 30 and 50 wt % of glasses exhibited significant hydroxyapatite (HA) formation at 72 h of immersion. The compression strength of the samples was also measured. The Young’s modulus was similar for the 10 and 30 wt % glass-containing scaffolds whereas it increased to 90 MPa for the 50 wt % glass containing scaffold. Upon immersion up to 72 h, the Young’s modulus increased and then remained constant for longer immersion times. The scaffold prepared could have great potential for bone and cartilage regeneration.

## 1. Introduction

In tissue engineering, the challenge lies in developing materials and scaffold geometries meeting all requirements to be used as a 3D template for bone regeneration. Indeed, it is common knowledge that optimal 3D scaffolds should be biocompatible. In the best case, not only would the materials be biocompatible, but they would also trigger cells to proliferate and differentiate to support further bone/tissue growth. Thus, for bone regeneration, the scaffold should be at the minimum osteoconductive and at best osteoinductive. The scaffolds should be biodegradable and leave no residues behind after complete degradation. However, the rate of degradation should be compatible with the rate of tissue regeneration. The mechanical properties should be tailored in order to meet standards for load bearing applications. The pore size and density should be tailored in order to allow for the migration of ions beneficial in the healing process, fluids and cells. The large pore size, porosity and the interconnectivity of the pores are of importance to ensure vascularization and tissue ingrowth. Finally, the scaffold should be easily re-shaped to match any defect size and shape [1].

Thus, the requirements for optimal scaffolds described above clearly suggest that one material will not be sufficient. Bioactive glasses are promising materials due to their proven ability to be osteoconductive and osteoinductive [2,3]. Bioactive glass has also the advantage of being tailorable, from a composition point of view. This allows for tailored dissolution rates and for the incorporation of ions with positive effect on human bone metabolism and angiogenesis [3,4,5]. However, it was demonstrated that the crystallization kinetics of the typical bioactive glasses 45S5 and S53P4 is not in favor of particles sintering [6]. Furthermore, it was demonstrated not only that the optimal sintering temperature for glass 45S5 led to significant glass crystallization, but that partial to full crystallization decreases the glass’ bioactivity in silicate glasses and even suppress it in phosphate-bioactive glasses [7,8,9]. In recent years, researchers have attempted to develop bioactive glasses with extended working range to facilitate the heat-induced sintering of particles or, more generally, hot forming [10,11,12]. One category of glass composition has emerged which shows promising thermal properties: borosilicate glasses [2]. Borosilicate glasses have been found to have thermal properties enabling sintering without inducing crystallization at relatively low temperature [13]. Furthermore, such glasses were found to dissolve at a faster rate than the silicate counterpart and to convert more efficiently into hydroxyapatite [13,14,15,16,17]. However, the inherent brittleness and hardness of glasses makes re-shaping of the scaffold challenging.

Thus, a composite containing a polymeric matrix and a bioactive glass in the borosilicate composition may be promising for tissue engineering applications. Poly(l-lactide-co-ε-caprolactone) (PLCL) is a biodegradable polyester synthetized by ring-opening polymerization. The l-isomer of the lactic acid is naturally present in mammalian biological systems and the use of ε-caprolactone in the copolymer greatly influences the polymers elastic properties. PLCL degrades by hydrolysis of its ester linkages in physiological conditions and is therefore an attractive polymer to be used in implantable devices [18,19]. PLCLs properties have been thoroughly studied [19,20] and both poly-ε-caprolactone and poly-l-lactide as homopolymers have been proven biocompatible and approved by the Food and Drug Administration (FDA) for a wide range of applications in the biomedical field [21].

In this context, we propose porous composite materials containing 10, 30 and 50 wt % of borosilicate bioactive glass powder embedded in a PLCL matrix. The porous scaffolds were obtained by using supercritical CO_2_. The impact of glass particles content on the material porosity and the polymer thermal properties was investigated. Dissolution tests were performed in simulated body fluid. At 6, 24, 48, 72, 168, and 336 h of immersion, the solution was analyzed in terms of pH and Ca ion content. The immersed scaffolds were dried and the presence of hydroxyapatite formation was assessed by FTIR. The mechanical properties of the scaffolds were assessed as a function of immersion time.

## 2. Materials and Methods

### 2.1. Composite Manufacturing

Borosilicate bioactive glass with composition 26.93SiO_2_-26.93B_2_O_3_-21.77CaO-22.66Na_2_O-1.72P_2_O_5_ (in mol %) was melted from batches containing mixtures of sand (99.4% pure SiO_2_), and analytical grades of Na_2_CO_3_, H_3_BO_3_, CaCO_3_, and CaHPO_4_·2H_2_O. The glass was melted in air at 1200 °C, casted into a graphite mold and annealed 40 °C below its glass transition temperature overnight. The glass was further crushed and sieved into particles with sizes between 125 and 250 µm. The glass was then mixed together with commercial biodegradable poly(l-lactide-co-ε-caprolactone) with 70% L (l-lactide)/30% CL (ε-caprolactone) copolymer (Purac Purasorb, Amsterdam, The Netherlands) in acetone. Glass particles, 10, 30 and 50 wt %, were then introduced to the polymer. The mixtures of dissolved polymer and borosilicate bioactive glass were stirred overnight or until most of the acetone had evaporated. The materials were then allowed to dry for 48 h under vacuum. After drying, the obtained pastes were then processed into rod-like compact pellets with a diameter of 12 mm. The dried composite pellets were made porous by supercritical CO_2_ (ScCO_2_) treatment and then cut into scaffolds of 3–4 mm in height.

### 2.2. Sample Analysis

Cross-sections of scaffolds were observed with an optical microscope at 20× magnification. A more accurate porosity analysis was conducted using X-ray micro-computed tomography (µCT). MicroXCT-400 (Carl Zeiss X-ray Microscopy, Inc., Pleasanton, CA, USA) was used with tube voltage 140 kV and current 71 µA. Pixel size was 5.6 µm. Porosity analysis was done with Fiji [22] using a BoneJ [23] plugin. µCT visualizations were done with Avizo 9.1 (FEI Visualization Sciences Group). The porosity, pore size and pore size distribution was quantified on one representative sample over a volume of 4 × 4 × 3 mm^3^.

The bioactive glass (BAG)—PLCL weight percent ratio between glass and polymer in the scaffolds were checked with thermogravimetric analysis (TGA; TGA Q500, TA Instruments, New Castle, DE, USA). 20 mg of samples were heated to 700 °C in air, in an Al_2_O_3_ pan, at a heating rate of 20 °C/min. The glass transition temperature (Tg) of the polymer was obtained using a differential scanning calorimeter (DSC; DSC Q1000; TA Instruments, New Castle, DE, USA). The heating rate was 20 °C/min, the cooling rate 50 °C/min and the temperature range was −50 °C to +200 °C. Nitrogen was used as the sweeping gas. All Tg were recorded at the inflection point of the first endothermic event. The reported Tgs correspond to the average of 3–4 parallel samples. In all measurements, the samples were heated twice. The first heating was performed to obtain the Tg of the composite as obtained after manufacturing, and the second to erase all difference in thermal history from sample to sample and obtain the Tg of the plain polymer. Both DSC and TGA results were analyzed using Universal Analysis Software.

### 2.3. Dissolution Test

Degradation tests were conducted on porous scaffolds (10 mm diameter and 3–4 mm height, as measured by means of a caliper) by soaking them in 1 mL of simulated body fluid (SBF) per 10 mg of sample mass for various time intervals (6, 24, 48, 68, 168 and 336 h). SBF buffer solution was prepared according to the protocol described by Kokubo and colleagues [24] at 37 °C and pH 7.4. Three to four parallel samples of each type of composition (according to the % wt of BAG) were placed in a shaking incubator at 37 °C. 

After each immersion time point, the pH of the SBF solutions was measured with calibrated a pH meter (Mettler Toledo, Columbus, OH, USA) at 37 °C. Five mL of the immersion solution was diluted in 45 mL of deionized (DI) water for atomic absorption spectroscopy (AAS) measurements (PerkinElmer AAnalyst300, PerkinElmer, Waltham, MA, USA). AAS was employed to quantify the amount of calcium ions in the solutions. After immersion, scaffolds were carefully wiped by gentle contact with a paper and weighed immediately for the determination of wet weight as a function of the immersion time. After that, samples were left to dry in ambient conditions for 1–2 days, then put to vacuum for at least 24 h before taking the dry weight. 

The change in the mass was calculated as

(1) $$\Delta m\left(\%\right)=\left(W_{dry}-W_{orig}\right)/W_{orig}\times 100\%$$

where *W_orig_* and *W_dry_* are the weights of dry sample scaffolds before and after the immersion, respectively.

Each test was repeated three to four times for each composition and results were expressed as average values.

The average molecular weight of the copolymer was analyzed with gel permeation chromatography equipped with both a refractive index and a UV–VIS detector (GPC; Shimadzu high performance liquid chromatography; HPLC, Lab Solutions GPC, Shimadzu Corp., Kyoto, Japan). HPLC-grade chloroform was used as a solvent and eluent. Sample size ranged from 8.3 mg (BAG 10% samples) to 15 mg (BAG 50% samples) in order to obtain 1.5 mg/mL of polymer in solution. Samples were dissolved overnight in 5 mL of chloroform. Samples were filtered twice through 0.45 µm syringe filter to remove the glass particles before being passed into a pre-column set at 40 °C and two mixed bed columns (Shodex GPC KF-806M, 8.0 mm [Internal Diameter] × 300 mm [Length]) connected to the GPC system. The injection volume of the sample was 100 µL and the flow rate of the eluent was 1.0 mL/min. Two parallel samples were analyzed from each sample scaffold (for each time point and composition) and the results were compared to the plain raw material PLCL. Results were analyzed with Lab solutions GPC Postrun Analysis Software.

The IR absorption spectra of the glass, the polymer and composites were recorded using Fourier transform infra-red spectroscopy (PerkinElmer Spectrum One FT–IR, PerkinElmer, Waltham, MA, USA) in attenuated total reflectance (ATR) mode. All spectra were recorded within the range 600–4000 cm^−1^, corrected for Fresnel losses and normalized to the absorption band showing the maximum intensity.

Finally, a compression test was performed on dry samples using an Instron 4411 mechanical tester (Instron, Norwood, MA, USA). A 500 N load cell was used with a crosshead speed was 1 mm/min.

## 3. Results

The aim of this paper is to analyze various ratio of glass/polymers in composite with potential in tissue engineering. The polymer should allow for easy shaping and formation of pores adequate for tissue ingrowth and vascularization, and the bioactive glass should improve the mechanical properties (especially Young’s modulus) while guarantying formation of hydroxyapatite.

**Figure 1:**
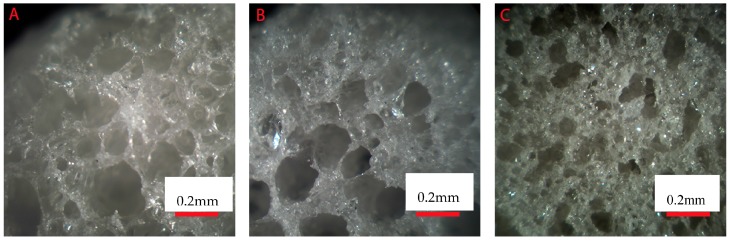
Optical microscope image of the scaffolds’ cross section: (**A**) 10; (**B**) 30 and (**C**) 50 wt % of borosilicate glasses.

After the processing of the composites, thermogravimetric analysis was conducted on the three scaffold compositions. In order to ensure accurate results, three samples from three scaffolds of each composition were analyzed. At the end of the thermal cycles, it was assumed that only the glass was left behind. The composite scaffolds were found to contain 11 ± 2, 33 ± 4 and 51 ± 4 wt % of bioactive glasses. Figure 1 presents the cross section of the three studied composite (10, 30 and 50 wt % of borosilicate bioactive glasses) scaffolds after processing. All scaffolds were found to present a high density of pores that appears to be interconnected. In all samples, the pore size distribution appeared broad. However, an increase in bioactive glass content led to materials with overall smaller pore size.

**Figure 2:**
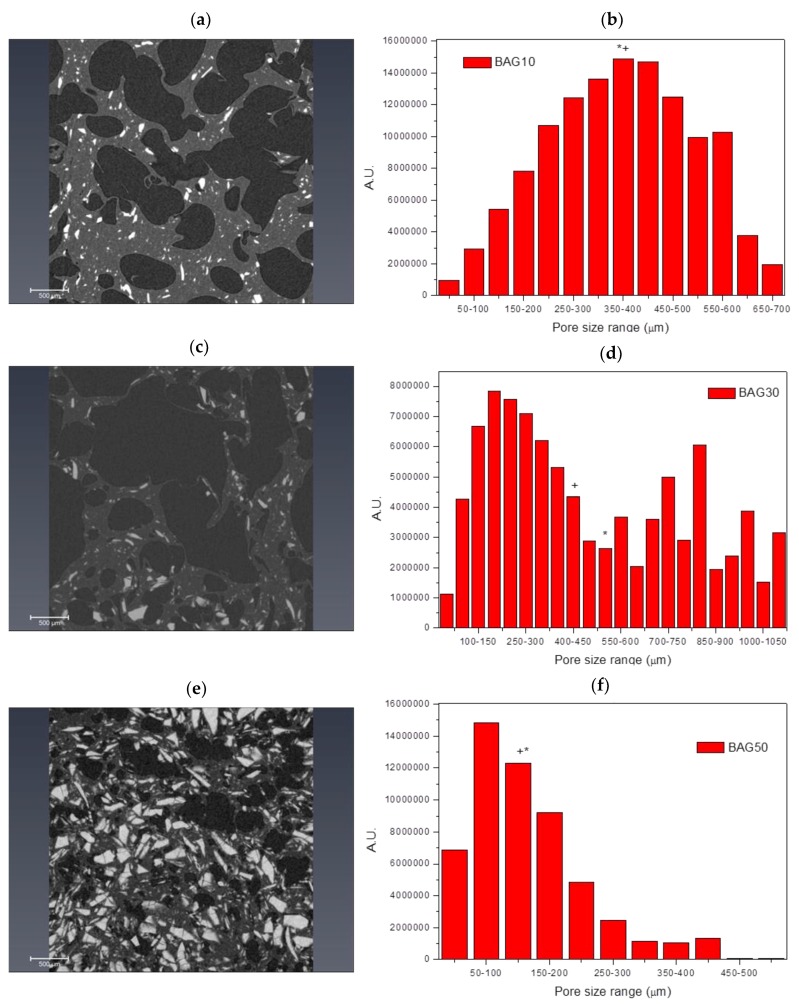
Microcomputational tomography image and pore size distribution of different bioactive glass (BAG) scaffolds by wt %: BAG10 (**a**,**b**); BAG30 (**c**,**d**) and BAG50 (**e**,**f**). (+ and * denote the average and median pore size, respectively).

Figure 2 presents the micro-computed tomography images and pore size distribution for the samples labelled BAG10 (a and b), BAG30 (c and d) and BAG50 (e and f). All samples produced a significant amount of pores. Pore analysis showed that samples containing 10 and 50 wt % of bioactive glass had a rather narrow pore size distribution compared to the sample containing 30 wt % bioactive glasses. The average pore size was found to be 375 ± 152, 523 ± 330 and 175 ± 104 μm for the BAG10, BAG30 and BAG50, respectively. The median pore size was found to be 372, 424 and 171 μm, respectively. Finally, the porosity was calculated and found to be 54 ± 5%, 64 ± 5% and 27 ± 5%, respectively.

Differential scanning calorimetry was performed on the prepared scaffolds. The glass transition temperature was recorded after 2 successive heating/cooling cycles. The first Tg obtained gives the indication of the thermal properties of the polymer after processing while the second Tg gives information about the polymer once the thermal/stress history of the material has been erased. The first Tg, noted later as Tg_1_, was found to increase from 17.4 ± 1.1 to 20.6 ± 1.9 and to 22.3 ± 1.4 °C when the glass content increased from 10 to 30 and to 50 wt %, respectively. The second Tg, noted as Tg_2_, was found to be constant, regardless of the glass content, at 22.4 ± 1.0 °C.

**Figure 3:**
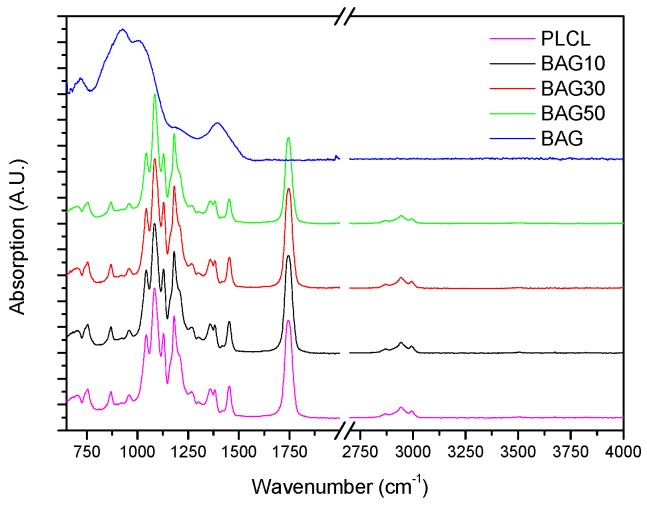
FTIR–ATR spectra of the investigated scaffolds (BAG10, BAG30 and BAG50) and of the borosilicate bioactive glass (for comparison).

Figure 3 presents the FTIR spectra of the samples under investigation, along with the FTIR spectra of the glass and PLCL. All spectra were background corrected and normalized to the peak with maximum intensity. The FTIR spectra of all composites presented peaks in the 2750–3100 cm^−1^ region as well as numerous peaks in the 600–1800 cm^−1^ region. As a comparison, the FTIR spectra of the bioactive glass is also presented. Broad bands characteristic of amorphous materials can be seen in the 600–1600 cm^−1^ region.

The dissolution/reaction test was performed in simulated body fluid (SBF). The goal was to evidence the formation of hydroxyapatite at the surface of the scaffold upon immersion, and the dissolution of the glass. The complete dissolution mechanism of the bioactive glass has been discussed elsewhere. It is known that upon reaction in SBF, the borosilicate bioactive glasses induce precipitation of hydroxyapatite (HA). Change in pH, and ions released in solution are good indications of the glass degradation.

**Figure 4:**
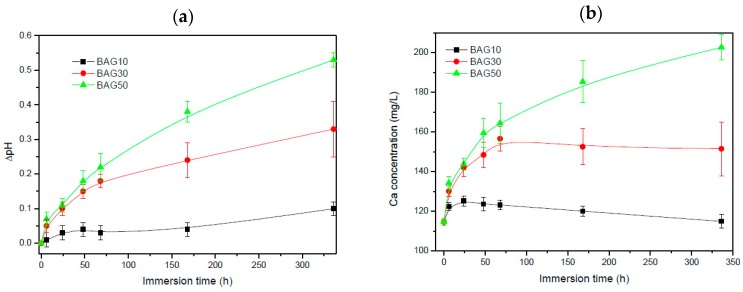
Change in pH (**a**) and in Ca concentration (**b**) in the simulated body fluid (SBF) solution as a function of immersion time.

Figure 4 presents the pH of the solution (a) and the Ca concentration in the solution (b) as a function of immersion time. All scaffolds exhibited an increase in pH and Ca concentration with increasing immersion time. Such an increase is more pronounced with increasing the glass content in the scaffolds. 

**Figure 5:**
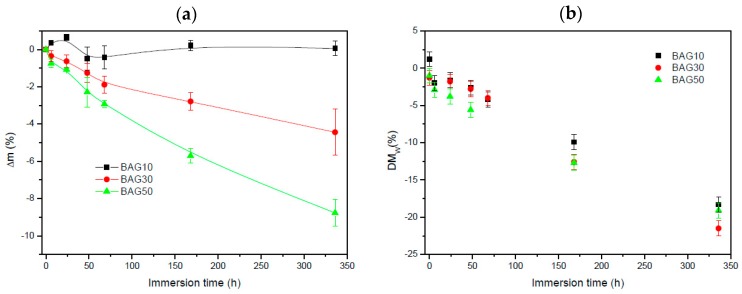
Change in sample mass (**a**) and the polymer molecular weight (**b**) as a function of immersion time.

Figure 5 exhibits the change in sample mass (a) and the polymer molecular weight (b) as a function of immersion time. Within the accuracy of the measurements, it appears that the change in mass of the composite containing 10 wt % of BAG was negligible. With an increase in BAG content, the mass loss increased. The molecular weight of the polymer (Figure 5b) was found to slightly decrease (almost linearly) for all samples as a function of immersion time. However, no clear trend, as a function of BAG content, could be extracted and it is assumed that all samples behaved in a similar manner.

**Figure 6:**
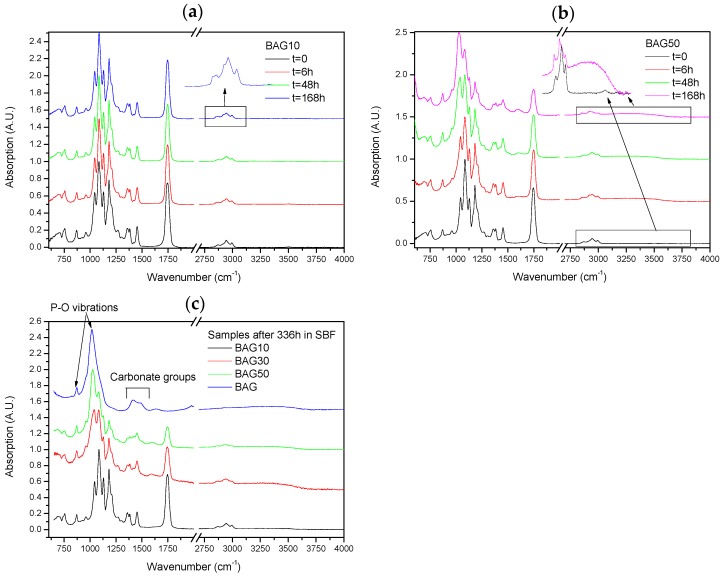
FTIR–ATR spectra of the composites labelled BAG10 (**a**) and BAG50 (**b**) at various immersion times and of all composites immersed for 336 h (**c**). In addition, the FTIR spectra of immersed bioactive glass alone in SBF for one week has been added Figure 6c to show the formation of the HA layer at the surface of the glass particles.

Figure 6 presents the FTIR–ATR spectra of the composites labelled BAG10 (a) and BAG50 (b) at various immersion times, and of all composites immersed for 336 h (c). With increasing the immersion time, the absorption peak’s shape and intensity remained approximately the same for the composite BAG10. The only minor changes noticeable can be seen in the insert of Figure 6a. While the original composite presented 3 bands at 2990, 2945 and 2860 cm^−1^ attributed to an alkyl group in the PLCL copolymer [25], the composites immersed in SBF all presented extra peaks at 2917 and 2850 cm^−1^, attributed to a –CH aliphatic structure (open chains). In Figure 6b the changes are more evident. With increasing the immersion time, the peaks related to the polymer structure decreased in intensity (Figure 6b). A peak at 1025 cm^−1^ was found to increase in intensity with increasing immersion times. Similarly a broad band in the 3200–3700 cm^−1^ region arose and increased in intensity with increasing immersion time. At 168 h the –CH peaks at 2945 and 2860 cm^−1^ almost disappeared at the expense of the absorption bands at 2920 and 2850 cm^−1^. Lastly after 368 h of immersion (Figure 6c), the FTIR spectra of composite BAG10 still appeared unchanged compared to the untreated composite. With increasing the BAG content and immersion time, one can note the increase in the band at 1025 cm^−1^, as well as its sharpening. The intensity of the absorption bands related to the decrease in polymer intensity. For comparison the FTIR spectra of the BAG alone immersed for such duration is provided. All the peaks were found to be attributed to carbonate and phosphate vibrations due to the conversion of the BAG into hydroxyapatite.

**Figure 7:**
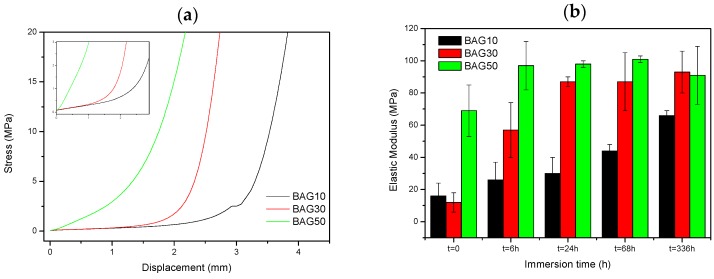
Typical compression test curves (**a**) and Young’s modulus of the composites as a function of immersion time (**b**). The insert in Figure 7a aims to show the initial linear relationships between stress and strain, where the Young’s modulus was quantified.

Figure 7 presents the typical curve obtained during compression testing (a), as well as the evolution of the Young’s modulus of the composite with regards to the scaffold immersion time (b). From Figure 7a, the stress/strain curve is typical of the curve obtained for porous materials [26]. Typically, it is well accepted that porous materials present three distinct domains in the compressive stress/strain curve. First we should see a linear elastic region then a plateau corresponding to the collapse region and then an abrupt increase in the stress due to the densification. In our sample, the plateau could only be seen for the sample BAG10. For all materials, the slope of the linear elastic domain (shown as an inset in Figure 7a) was measured. Upon testing, no maximum compression stress (sample fracture) could be measured. From Figure 7b, the slope of the initial deformation increases with increasing BAG content. With increasing immersion time (up to one week), the slope of the initial deformation linearly increases for the sample containing 10 wt % of BAG (BAG10). BAG30 and BAG50 exhibit an increase in the elastic modulus following an immersion of up to 24 h and 6 h, respectively. For both samples, it appears that their moduli reach a maximum (90 ± 10 Mpa) and remain constant for longer immersion times.

## 4. Discussion

**Figure 8:**
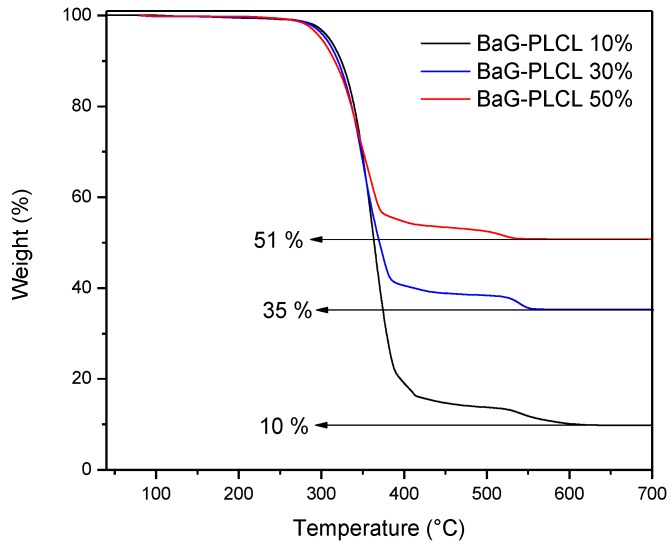
TGA thermogram of the composites under investigation.

The amount of glass in each sample was measured by means of TGA (Figure 8). The glass content was found to be 11 ± 2, 33 ± 4 and 51 ± 4 wt % for the 3 composites under investigation. This is in agreement with the desired BAG/polymer ratio. 

This suggests good distribution of glass particles within the polymer matrix during processing. The introduction of 50% borosilicate bioactive glasses led to denser scaffolds with smaller pores size (Figure 1 and Figure 2). While the pore size remained large enough to suspect proper cell migration within the pores, and further vascularization, the porosity of the scaffold is too low for any practical application. Samples labelled BAG10 and BAG30 demonstrated a large median pore size, interconnected porosity and overall porosity larger than 50%, which is promising for composite scaffold manufacturing. 

The increasing amount of glass within the scaffolds also led to an increase in the glass transition temperature (Tg_1_). Such an increase in Tg could not be attributed to a change in the polymer structure during manufacturing as, upon a second heating cycle, the Tg of the polymer was identical in all scaffolds tested. Thus, it is believed that the increase in Tg_1_ is due the increased affinity between the filler (BAG) and the polymer matrix, as reported in Al_2_O_3_ containing epoxy [27,28]. To ensure that the structure of the polymer remained un-altered, FTIR–ATR was performed. Figure 3 presents the FTIR spectra of all the investigated samples, along with the FTIR–ATR spectra of the glass and the PLCL alone. One can see that the FTIR spectra of the composite exhibited solely the absorption vibration related to the PLCL polymer. Upon immersion in SBF, the mass of the BAG30 and BAG50 composites decreased significantly (Figure 5a), whereas the mass of the sample BAG10 remained almost constant over the two weeks’ immersion time. The change in mass is unlikely to be due to the polymer degradation. Indeed, despite the significant decrease in the average molecular weight of the polymer, as a function of immersion time, all scaffolds exhibited similar molecular weight decreases. Thus, the change can be better correlated to the borosilicate glass dissolution upon immersion in SBF. The reduction in the glass particle size could also, ultimately, lead to a loosening of the glass particles and thus to a sharp change in the sample weight. Furthermore, the glass transition temperature of the composite was recorded as a function of immersion time (not shown here). The Tg_1_ was found to remain constant over the two weeks of immersion for the sample BAG10. However, the Tg_1_ was found to decrease to 16.1 ± 1.1 °C for both BAG30 and BAG50. This may confirm a loosening of the glass particles (due to the glass reaction with the aqueous solution), releasing some of the stress induced by the presence of the glass in the polymeric matrix. It is interesting to note that the Tg_2_ (after a second heating cycle to remove the polymer history) in all samples and regardless of the immersion time was at 22.4 ± 1.0 °C. This indicates that only the polymer chain length is modified, whereas the chain structure does not seem to be greatly impacted.

The dissolution/reaction of the glass in the solution (as a function of immersion time) was also studied. From Figure 4a, all scaffolds exhibited an increase in the pH with increasing immersion time. The increase in pH is more obvious when the bioactive glass content was increased. Such an increase in pH is typical from bioactive glasses. This is generally attributed to the leaching of the alkaline and alkaline earth ions from the glass to the solution [29]. This is further confirmed in Figure 4b where the Ca concentration in the SBF was found to increase with an increase in the glass content in the scaffolds, and in immersion time. However it is noted that presence of boron tended to raise the pH to higher values than typical silicate based glasses [7,13]. Furthermore, an increase in pH to values higher than 8 have been found to have negative effect in cell differentiation [30]. An excess of boron within the culture solution was also found to have negative effect on the proliferation and differentiation of cells [13,31,32]. However, in this study, not only was the boron content reduced, compared to the base glass alone, but the pH raise was maintained below the critical threshold of pH8. 

Figure 6 presents the FTIR–ATR spectra of the scaffolds BAG10 (Figure 6a) and BAG50 (Figure 6b) as a function of immersion time. The spectrum obtained from the BAG10 scaffolds does not reveal major changes, except in CH vibration (within the 2900–3000 cm^−1^ region). As expected, all untreated scaffolds presented vibration at 2995 cm^−1^ related to CH_3_ vibration in polylactic acid (PLA) and at 2945 and 2870 cm^−1^ related to CH_2_ vibration in PLA and polycaprolactone (PCL) [33]. Upon dissolution, the bands at 2945 and 2870 cm^−1^ should not change, as reported in [34] for the PCL degradation, and the band at 2995 cm^−1^ should disappear [33]. However in our case, upon dissolution the bands at 2995, 2945 and 2870 cm^−1^ decreased in intensity while two new bands at 2920 and 2860 cm^−1^ appeared. In such regions, these vibrations can also be attributed to CH vibration. The reason for the change in CH vibration is not yet well understood but it is likely due to an interaction between ions from the glass, or glass dissolution by-products that would lead to change in the energy required to allow the vibration of the CH_2_ groups. While no drastic change in the spectra could be seen in BAG10 as a function of immersion time, significant changes could be seen in BAG50. While all bands related to the polymer decreased in intensity, a broad band at 1023 cm^−1^ increased in intensity, sharpened and shifted to higher wavenumbers. The presence of the shoulder at 959 cm^−1^ may be attributed to C–O vibration modes in CO_3_^2−^, and to P–O–P bonding [35,36]. This is further confirmed by the appearance of the shoulder at 875 cm^−1^ that can be attributed to P–O vibration [36]. It is generally accepted that the presence of the vibration bands at ~1020 cm^−1^ along with a vibration band at 875 cm^−1^ can be attributed to Ca deficient HA [37]. This was further confirmed in [38] where it was demonstrated that scaffolds from the bioactive glass used in this study precipitated an HA layer with a Ca/P ratio close to 1.67. The Figure 6c presents the spectra of all scaffolds and of the corresponding bioactive glass particles immersed for 336 h. With an increase in the the bioactive glass content within the scaffolds, the signal of the HA layer at the surface of the scaffold was enhanced. Indicating that more HA was covering the scaffolds. 

Finally, the mechanical properties of the scaffolds have been tested in compression. The typical stress-strain curve obtained for the three scaffolds are shown in Figure 7a. No fracture of the samples could be evidenced. The elastic modulus was calculated (Figure 7b) and found to increase with increases to the bioactive glass content in the scaffolds. With increasing the immersion time, the elastic modulus increased almost linearly for the composite BAG10, whereas for composites BAG30 and BAG50, the modulus reached a maximum after 6 and 24 h of immersion, respectively. Typically a decrease in the elastic modulus is expected due to the opening of the pores. However, in this case the rapid transformation of the borosilicate into HA may have filled the pores and enhanced the mechanical properties. Karageorgiou and Kaplan reported that the elasticity of cortical bone (midfemoral) was around 17 GPa, whereas trabecular bone from the proximal femur showed a modulus of 0.4 GPa [39,40,41]. However, one should keep in mind that if tested wet, the modulus is likely to further decrease [42]. Therefore, while the composite containing 10 wt % of bioactive glass does not present a great interest, the samples containing 30 and 50 wt % of bioactive glass could have potential in non-load-bearing application, especially since both constructs stiffen within 6–24 h of immersion.

## 5. Conclusions

PLCL porous scaffolds with 10, 30 and 50 wt % of borosilicate bioactive glass were successfully produced using super-critical CO_2_. The presence of the bioactive glass was found to not interfere with the degradation of the polymer. However increasing glass content reduced the overall pore size and concentration, as well as the overall porosity. Clear signs of hydroxyapatite formation could be seen using FTIR at the surface of the composites processed with 30 and 50 wt % of BAG. Furthermore, the mechanical properties of the scaffolds were analyzed in compression and as a function of immersion time. The modulus of all materials increased with increasing immersion time. For the samples BAG30 and BAG50, the modulus reached a maximum at 24 and 6 h of immersion, respectively. The increase in modulus could be attributed to the formation of hydroxyapatite within the scaffolds’ pores. 

The scaffolds containing 30 wt % of BAG seemed to be the most appropriate material for use in tissue engineering, as the pore size and pore volume remained high. Furthermore a clear sign of hydroxyapatite could be seen after a few days of immersion in SBF. This material could be a good candidate for bioactive scaffolds in bone regeneration, especially as the pH increase was within the limits tolerated by cells, and the boron release should remain at a non-toxic level.
